# The Dental Protective Association

**Published:** 1889-01

**Authors:** 


### THE DENTAL PROTECTIVE ASSOCIATION.

No step that our specialty has made since it merged from the position of a trade seems to us to have so important a bearing upon the future status of Dentistry as have the efforts at organization and self-assertion made during the past year. The organization and incorporation of the Dental Protective Association in Chicago is a movement in the right direction. We see vast possibilities for good in such an organization.

It is not the aim of the Dental Protective Association to organize to defeat legitimate and honorable business; but, on the other hand, to encourage and stimulate fair and open competition in the manufacture of all the necessaries that are used in the practice of dentistry. Judiciously handled, the money that it is proposed to collect can be used in many ways to fulfill these ends.

It is not our purpose in this article to specify any particular line in which abuses have been practised in the past. All are too well acquainted with cases where the name extortion could be applied to need mention from us. Neither do we mean to make open war against legitimate business enterprise; but only in those cases where marked injustice and oppression have not only been practised, but with a "What are you going to do about it" air that is particularly galling. Chains are not so hard to bear when their links are smoothed; and they are even used as ornaments and worn with pleasure at times. It, however, makes a very considerable difference as to who by, and the manner in which, the key is turned that binds for the time being our hands.

We do not desire to be understood as impugning the motives or business integrity of the parties who hold the control of certain necessities. They have become possessed of these patents, so far as we know, by fair and legitimate means. But what we do mean to say is, that they should never have been allowed to come into the possession of such claims, or, having obtained them, should be made to so use them as not to abuse their privileges.

We have no doubt that if the claims of quite a number of the existing patents on dental goods were to be contested at the present time, with the evident tendency of the courts to decide in favor of the people, very many of them would be declared invalid. But that is not the aim for which the Dental Protective Association was founded. Its sole purpose, as set forth in their appeal, which may be found in our advertising columns, is to prevent abuses, and the good sense of the profession will never allow the funds in its hands to be used to interfere with legitimate business enterprises. Among the many ways in which such a fund might be used is the purchasing of patents on desirable and useful inventions, and giving them to the profession, instead of allowing them to pass into the hands of large corporations and be shelved. The smaller houses would then be stimulated, and open competition would be encouraged. No fact, at the present time, is more patent than that the dental manufacturer and dealer is sadly in need of the right to manufacture a few of the leading staple articles that are in daily use in the office. This is not because such articles are not being invented, but because that as fast as they are brought out they are gobbled up and put to one side. Smaller houses are afraid to purchase them because of the certainty of purchasing a law suit.

It is a well known fact that no patent is considered of any value until it has been successfully defended in the courts. We are in favor of legitimate business all the way through; and once have fair competition established, and the price of dental goods will go in the same lines as that of other manufactured products.

But how is the Dental Protective Association going to bring this about? In many ways. In the first place, with a large fund at its command it can fight abuses which deserve to be overthrown; then again, it can purchase from first hands desirable patents and give them to the trade. They can then be manufactured by anyone who desires to enter into the business, and compete openly with other manufacturers; and, further, it can purchase existing patents from those manufacturers already holding them, at fair prices, and thus relieve the profession of the existing royalty which, if paid individually, amounts to an imposition in many instances. The refusal of any manufacturer or corporation to sell at an appraised valuation, would be evidence upon the face of it, that the special line of goods so manufactured was not sold at a fair legitimate profit. There should not be any antagonism between manufacturers and the profession. If the latter organizes and talks business it should not be decried. It surely has some rights besides paying out all its hard earnings to the manufacturer.

We have specified no names, and have only spoken in a general way. The time is not ripe for entering into the minutiæ of the question. The thing for the profession to do is to organize and get together a fund that will ensure respect and fear.

The character of the officers of the Dental Protective Association is above reproach. They are known to be men who have the courage of their convictions, and who have the interests of the dental profession at heart. They are not inexperienced, and have gone into this movement with the full recognition of the immense amount of labor attached to it. The fact that the funds are to be in the hands of such a prominent and well known man as Lyman J. Gage will give confidence in the movement, and we predict a hearty reception of it at the hands of the profession.

